# Resection of Thoracic Plasmacytoma and Corpectomy Through the Anterolateral Thoracic Approach: A Case Report

**DOI:** 10.7759/cureus.50627

**Published:** 2023-12-16

**Authors:** Jorge Del Pino-Camposeco, Eliezer Villanueva-Castro, Daniela Deustúa-Hernández, Obet Jair Canela-Calderón, Ernesto Martínez-de la Maza, Juan Nicasio Arriada-Mendicoa, Juan Antonio Ponce-Gómez

**Affiliations:** 1 Department of Neurosurgery, Instituto Nacional de Neurología y Neurocirugía Manuel Velasco Suárez, Mexico City, MEX; 2 Medical Education, Facultad de Medicina Benemérita Universidad Autónoma de Puebla, Puebla, MEX

**Keywords:** primary spine tumor, thoracic approach, sins score, multiple myeloma, solitary bone plasmacytoma

## Abstract

This case report details the case of a 57-year-old male who initially manifested low back pain radiating from the lumbar region to the left leg. Progressive symptoms included paresthesia on the plantar surfaces of both feet and gait instability attributed to weakness in the pelvic limbs. Computed tomography imaging revealed osteolytic lesions in the T9, T10, and T11 vertebral bodies, resulting in compression of the spinal cord. Subsequent contrast-enhanced magnetic resonance imaging validated these findings, confirming the presence of an extradural tumor. In accordance with the Spinal Instability Neoplastic Score (SINS), the case was categorized as indicative of potential spinal instability. Consequently, a surgical intervention was performed to excise the lesion. Thus, the role of SINS played a pivotal role in guiding the decision-making process for the chosen treatment modality.

## Introduction

Multiple myeloma (MM) is a malignant neoplasm that originates from B cells and gives rise to destructive osteolytic bone lesions. Among the complications frequently observed, a notable one is the pathological fracture of the vertebral body, which can lead to compression of the spinal cord. This particular complication affects around 5% of individuals diagnosed with MM [1]. Plasma cell neoplasia ranks as the second most prevalent hematologic malignancy, following non-Hodgkin lymphoma. It represents 1% of all cancers and approximately 10% of all hematologic malignancies. The two subtypes of plasmacytoma are solitary bone plasmacytoma and extramedullary plasmacytoma [2].

Approximately 5% of all plasma cell disorder cases are solitary bone plasmacytoma, with a male-to-female ratio of 2:1. Solitary bone plasmacytomas comprise 70% of all solitary plasmacytoma cases and mainly occur in the bones of the axial skeleton containing red marrow. The optimal treatment for solitary bone plasmacytoma of the spine remains controversial. Solitary bone plasmacytoma is highly sensitive to radiation therapy, and clinical trials have confirmed high response rates (60-80%) to radiation therapy. Decompressive surgery is indicated in the case of neurologic compromise due to spinal cord compression [3]. However, progression to MM has been reported in some patients with solitary plasmacytoma after initial radiation therapy [1,4].

Decision-making regarding augmentation, decompression, and stabilization in patients with spinal plasmacytomas is controversial. The Spinal Instability Neoplastic Score (SINS) may play a role during the decision-making process. Vertebral augmentation surgery can be performed in patients with painful spinal plasmacytomas with osteolytic changes with or without a fracture (SINS <13). Decompression and stabilization surgery are the treatments of choice in patients with SINS >12 [5].

This report aims to describe the anterolateral thoracic approach as a therapeutic measure for this type of lesion through an analysis of this type of tumoral pathology. Simultaneously, it aims to analyze and demonstrate the significance of the SINS as a scale for guiding therapeutic decisions in spinal tumors.

## Case presentation

A 57-year-old male with no previous personal medical history had paresthesia in the soles of his feet. One month later, this progressed to the involvement of both pelvic limb territories. Shortly thereafter, he reported gait instability due to weakness in both pelvic limbs. Two months later, he developed hypoesthesia at the level of the umbilical scar and a progressive loss of strength, making walking impossible. Ultimately, one month later, he reported difficulty urinating and constipation. Subsequently, he was referred to our hospital.

On presentation at our service, a physical examination revealed 5/5 strength in the thoracic limbs and 3/5 proximal and distal strength in both pelvic limbs. Deep tendon reflexes were increased in the lower limbs (+++), while the rest were normal. Sensation showed exteroceptive and proprioceptive hypoesthesia starting from T10. Gait assessment was not possible due to weakness. Tone and trophism were preserved, and a bilateral positive Babinski sign was present.

**Figure 1:**
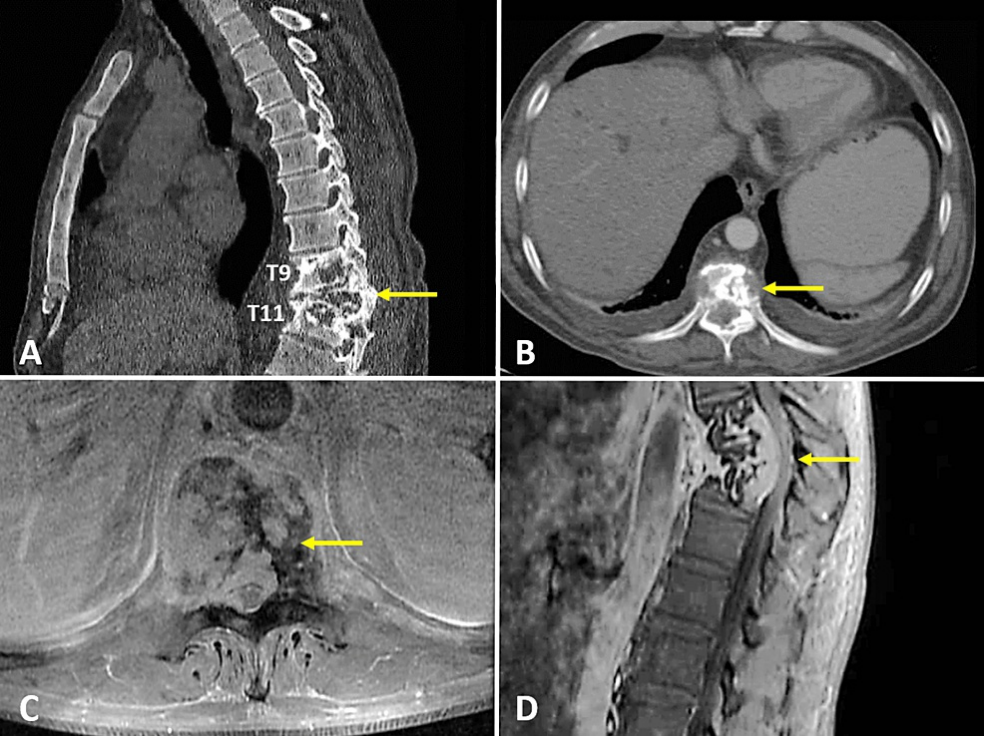
Preoperative imaging studies (A) Sagittal CT section with osteolytic lesions at the levels of T9, T10, and T11, with the presence of almost complete vertebral collapse of T10 and increased thoracic kyphosis (yellow arrow); (B) CT axial section at T10 level with osteolytic lesions (yellow arrow), which partially invade the spinal canal and the posterior column of the vertebra; (C) Axial contrast-enhanced MRI at the T10 level with the presence of a heterogeneously contrast-enhancing tumor lesion with total occupation of the vertebral body and invasion of the vertebral canal (yellow arrow); (D) Sagittal contrast-enhanced MRI with a tumor lesion with anterior and posterior components to the vertebral bodies of T9, T10, and T11 with compression of the spinal cord (yellow arrow).

A CT scan was performed, which reported osteolytic lesions in the T9, T10, and T11 vertebral bodies with soft tissue formation causing pathological fracture of the T10 vertebral body and spinal cord compression of more than 50% (Figure 1A, 1B). A contrast-enhanced MRI was conducted to confirm the findings from the CT scan. Additionally, the MRI revealed diffuse enhancement in the posterior elements and confirmed the presence of an extradural tumor in T9, T10, and T11 (Figure 1C, 1D). 

The SINS was applied with a total score of 14 points (Table 1), signifying spinal instability. Consequently, based on the recommendations of the SINS, decisions were made, and a surgical procedure was performed. Regarding the surgical intervention, the procedure was performed in two surgical stages.

**Table 1:** SINS values of the patient The SINS values are based on reference values given in Fisher et al., 2023 [6] SINS: Spinal Instability Neoplastic Score

SINS Component	Patient Score
Location	
Semirigid (T3-T10)	1
Pain	
Yes	3
Bone lesion	
Lytic	2
Radiographic spinal alignment	
Deformity (kyphosis/scoliosis)	2
Vertebral body collapse	
>50% collapse	3
Posterolateral involvement	
Bilateral	3
TOTAL	14

First surgical time/transthoracic approach

With the patient placed in the left lateral decubitus position, a thoracotomy was performed via the sixth intercostal space to access the thoracic cavity. Once in the cavity, the lung was mobilized, displacing and releasing the pulmonary ligament. After identifying the esophagus and the descending aorta, at the level of T10-T12, the radiculomedullary arteries and the venous drainage of this area were released. Once the vertebral bodies were identified, the territory was ready to continue with the spinal portion of the surgical procedure. The affected vertebral bodies were identified, and discectomy was performed at T8-T9 and T11-T12, along with corpectomy of T9, T10, and T11 vertebral bodies. Hemostasis was ensured, followed by the insertion of a 40-FR endopleural tube and the placement of an expandable obelisk-type cage from T9 to T11. Subsequently, rib fracture stabilization, lung inflation, and closure of the thoracic cavity were completed.

Second surgical time

**Figure 2:**
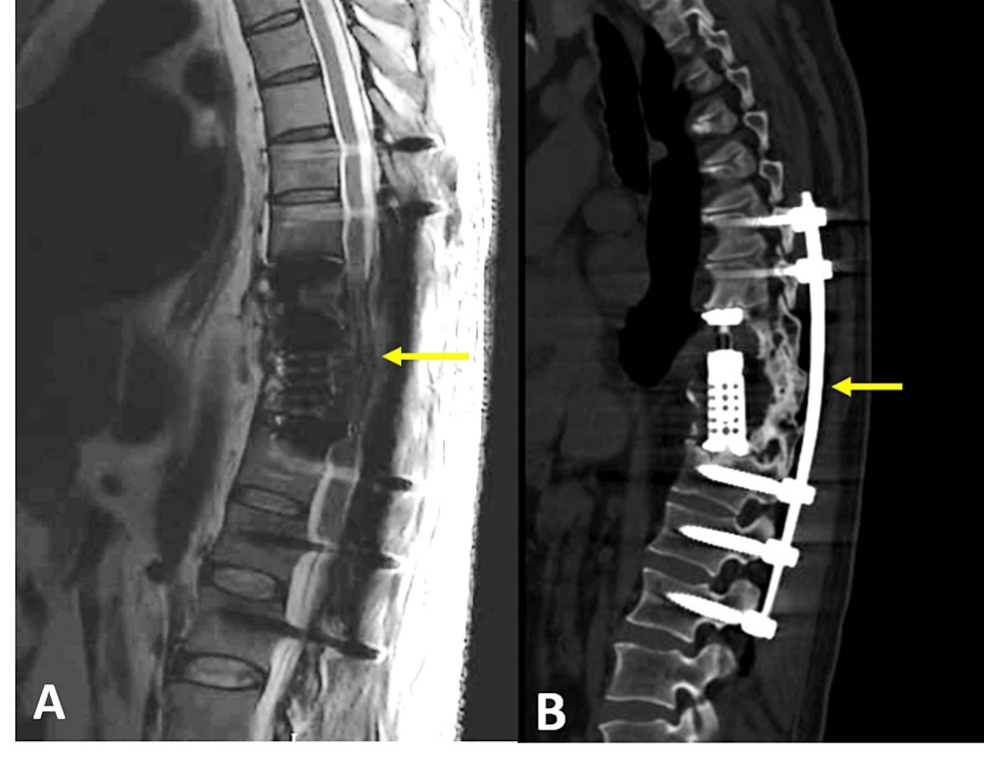
Postoperative imaging studies. (A) Sagittal T2 magnetic resonance imaging with adequate spinal decompression and restoration of thoracic kyphosis and vertebral height (yellow arrow); (B) Sagittal computed tomography showing adequate placement of the transpedicular screws and expandable cage (yellow arrow).

With the patient in the prone position and guided by fluoroscopy, paramedian incisions were made to facilitate the placement of Kirschner-type guides in the pedicles of the T9, T10, T11, T12, and L1 vertebrae, followed by the insertion of transpedicular screws and two bars (Figure 2). The ports were then removed, and closure of the paramedian incisions was achieved through fascial and skin suturing. An additional incision was made along the T9, T10, and T11 lines, and dissection was performed through the paraspinal muscle planes until the spinous processes were identified. A laminectomy was conducted using a Lexcel rongeur until the dural sac was exposed, revealing a vascularized, grayish, friable consistency lesion, which was completely excised.

Follow-up

The pathological report stated epidural plasmacytoma, bone without neoplastic infiltration, positive Kappa antigens, and negative lambda. One month after surgery and the beginning of physical rehabilitation, the patient arrived in a wheelchair. Upon physical examination, the patient exhibited muscular strength of 4-/5 proximal and distal in the lower limbs. The patient achieved standing with support but was unable to walk. Additionally, the patient received radiotherapy and chemotherapy in another external hospital; the chemotherapy regimen and dose of radiotherapy used are unknown. 

Six months after the patient arrived, he was walking independently. During the physical examination, there was a muscular strength of 4/5 in the lower limbs. The patient achieved standing and walking. Currently, at 20 months of follow-up, the patient presents with a muscular strength of 5/5, a preserved gait, and no evidence of tumor recurrence.

## Discussion

Solitary bone plasmacytomas are common primary malignant tumors of the vertebrae; they are defined by the presence of a single osteolytic lesion due to monoclonal plasma cell infiltration, with or without soft tissue extension [7]. The solitary bone plasmacytoma mostly affects vertebral bodies, and the most common location is in the thoracic region. Back pain is a common clinical feature and a variable neurological deficit due to lesion compression [8,9]. In the case presented, our patient had no pain; he had a motor deficit due to weakness in both pelvic limbs.

Diagnostic criteria for solitary bone plasmacytoma include a pathologically proven solitary lesion, normal bone marrow with no evidence of clonal plasma cells, a normal skeletal survey and MRI (or CT) of the spine and pelvis (except for the primary solitary lesion), and the absence of end-organ damage [3]. This case meets three of the four criteria for the diagnosis of solitary bone plasmacytoma; there was no evidence of normal bone marrow.

Maintaining or restoring spinal stability and achieving local disease management are the main goals of treatment for spine solitary plasmacytoma. Currently available forms of treatment are radiotherapy, surgery, vertebroplasty or kyphoplasty, and a mix of surgery and adjuvant radiotherapy [10]. It has been shown that even in situations when spinal cord compression is present, tumor removal can result in local disease management. In some cases, decompression has been demonstrated to maintain neurological function. In situations where discomfort is ascribed to fractures of the vertebral bodies, surgical decompression may also be advantageous. It is recommended to classify the patient's spinal instability using the SINS in order to choose the best surgical course of action [11].

The Spine Oncology Study Group (SOSG) created the SINS in 2010 as a tool for preoperative evaluation of spinal instability in patients with neoplastic spine illness. The purpose of the SINS is to assist the surgeon in determining treatment decisions for patients who suffer from spinal instability [12]. The SOSG has defined spinal instability as "the loss of spinal integrity as a result of a neoplastic process that is associated with movement-related pain, symptomatic or progressive deformity, and/or neural compromise under physiological loads” [11].

Six criteria are used by the SINS to assess vertebral mechanical instability: the location of the lesion, the nature of the pain, the type of bone lesion, the degree of vertebral destruction, the radiographic and spinal alignment, and the involvement of the posterolateral spinal elements. A final score is obtained by adding the ratings assigned to each parameter [13]. There is a minimum score of 0 and a maximum score of 18. Three categories of stability are derived from the overall score: possibly unstable (7-12 points), unstable (13-18 points), and stable (0-6). Furthermore, it is possible to examine the SINS as a binary indicator of the state of surgical referrals: stable (0-6) or "current or possible instability" (7-18 points). Patients scoring a 7 or higher are advised to contact a surgeon [11].

The SINS is a particularly trustworthy evaluation instrument. Because treating metastatic spine disease requires interdisciplinary expertise, proper SINS use is crucial [11]. Ramazanoğlu et al., based on the SINS scale to assess spinal instability associated with vertebral plasmacytoma, reported three patients with a SINS greater than 13 points in whom decompression and stabilization were performed with a good clinical outcome [5].

We present a case where a successful outcome was achieved in our patient with a total SINS of 14 (Table 1). This score was considered unstable, and we decided on initial surgical treatment to localize the tumor lesion, achieve spinal stability, and have tissue for histopathological diagnosis. Even though the posterior approach is the approach considered by most spine surgeons for spine tumors, due to the significant tumoral component of the lesion, the chosen procedure was to perform an anterolateral approach corpectomy on T9, T10, and T11, accompanied by the placement of an expandable obeliscPRO™ cage (ulrich GmbH & Co. KG, Ulm, Germany). Subsequently, a posterior approach laminectomy of T9, T10, and T11 was performed along with posterior instrumentation involving the placement of percutaneous transpedicular screws at T7, T8, T12, L1, and L2 (Figure 2).

The application of the SINS score is a useful tool in making decisions to treat spinal tumors. Decompression of the spinal tumor and stabilization have been shown to be of great benefit in the treatment of spinal instability, pain, and/or neurological deficits [14].

For local control of the tumor lesion, the R0 resection, which entails complete surgical removal of the tumor, would be optimal. It requires that there be no cancer cells visible at both the macro and microscopic levels. Given the anatomy of the area, this is a challenging task to accomplish in big spinal plasmacytomas. The goal should always be to remove as much of the tumor as feasible while maintaining the integrity of the surrounding tissues and achieving spinal decompression without causing harm to the spinal cord. Neoadjuvant radiation therapy is a possibility in certain big plasmacytomas in order to separate the tumor [15].

Within five years, at least 50% of solitary plasmacytomas will develop into MM if treatment is not received [16]. When used as the first line of treatment for solitary plasmacytoma, local irradiation offers good local control (85-90%), which may result in a long-lasting remission or even a cure [17]. Even at modest doses of radiation therapy, it was not able to stop the course of MM or stabilize the spinal column, even if it did achieve significant rates of local control. It is standard practice to utilize a dose of 40-45 Gy when treating with RT; nevertheless, it has been demonstrated that above 30-35 Gy, there is no dose-response association [17].

Baumgart et al. described that overall survival was longer with postoperative radiotherapy; however, there was no statistical significance for postoperative chemotherapy [2]. Our case received postoperative radiotherapy and has not recurred for 24 months, with an important clinical improvement.

## Conclusions

The surgical management of this type of spinal tumor continues to be controversial. Although there are lesions that have a good response to radiotherapy, there are reports in which progression to MM is reported. In some cases, such as those with instability, severe pain, or acute compression, surgical intervention might be required before radiotherapy. Regarding surgical management, there are several available options; however, the importance of applying the SINS relies on its role as a determinant of the relationship between spinal tumor instability and the need for stabilization surgery.
